# Restoring the Right Stream: Training-Free OOD Robustness for Vision–Language–Action Policies

**DOI:** 10.3390/e28090975

**Published:** 2026-09-02

**Authors:** Zhiyin Yan, Gang Shen

**Affiliations:** Smart Tower Co., Ltd., Beijing 100195, China

**Keywords:** vision–language–action models, out-of-distribution robustness, test-time adaptation, manifold projection, proprioception, robot manipulation, training-free

## Abstract

Vision–Language–Action (VLA) policies remain brittle under modest distribution shift. On LIBERO-Plus, contemporary models that solve clean tasks at high rates can fall below 30% success when the camera’s viewpoint or the robot’s initial pose is perturbed. Most training-free test-time remedies address this problem through the image stream, for example, by augmenting, purifying, or selecting visual observations. In our controlled evaluation, this family of methods improves mean success by only about three points and leaves the robot-initial-state failure largely unresolved. This paper studies the failure at the level of input streams. A VLA receives visual tokens, a proprioceptive state token, and language tokens; different perturbations can move different streams away from their training manifold. In particular, the robot-initial-state perturbation directly shifts the proprioceptive token; therefore, image-space interventions have limited leverage. We introduce Gated Per-Stream Manifold Restoration (G-PSMR), a training-free wrapper for a frozen policy. For each stream, a lightweight gate detects off-manifold inputs and applies a stream-specific restoration before the policy forward pass. We instantiate the framework with entropy-gated visual consensus and gated relative-orientation debiasing, which preserves the within-episode orientation trajectory. In the original 280-episode paired evaluation, the joint method improves total success by +5.3 points compared with a +3.2 image-only gain and raises the most fragile factor from 20% to 38%. On 1120 previously unevaluated, manifest-disjoint task instances, the state restoration improves robot-initial-state success from 23.8% to 28.7%; a separate prospectively specified confirmation on 600 new gate-active instances yields 26.3%→32.2% (+5.8 points; 95% CI [+3.2,+8.5]; p<0.001). Together, the original cross-stream results and two independent state-stream evaluations support the central principle of matching the restoration to the input stream carrying the shift.

## 1. Introduction

Vision–Language–Action (VLA) models are becoming a standard foundation for generalist robot manipulation. They typically start from a pretrained vision–language backbone and fine-tune it to map images, language, and proprioception to robot actions [1,2]. Their performance in nominal benchmark settings is now high, but their robustness is much less settled. On the recent LIBERO-Plus benchmark [3], contemporary VLAs such as OpenVLA, OpenVLA-OFT, and $\pi_{0}$ can drop from about 95% success to below 30% when the camera’s viewpoint or the robot’s initial pose is perturbed. This gap matters for deployment, where collecting new demonstrations and retraining a policy for every shift is rarely practical. Training-free test-time methods therefore offer an appealing alternative: the policy remains frozen, and robustness is added only around the inference procedure.

The current test-time toolbox, however, concentrates on a narrow part of the policy input. Most methods modify the image stream or select among action outputs derived from visual variants. In our original paired evaluation, the strongest image-space configuration improves mean success by +3.2 points, yet the robot-initial-state factor remains the largest unresolved failure mode. This pattern motivates a complementary question: which input stream carries each shift, and can an intervention be applied directly to that stream?

**Figure 1:**
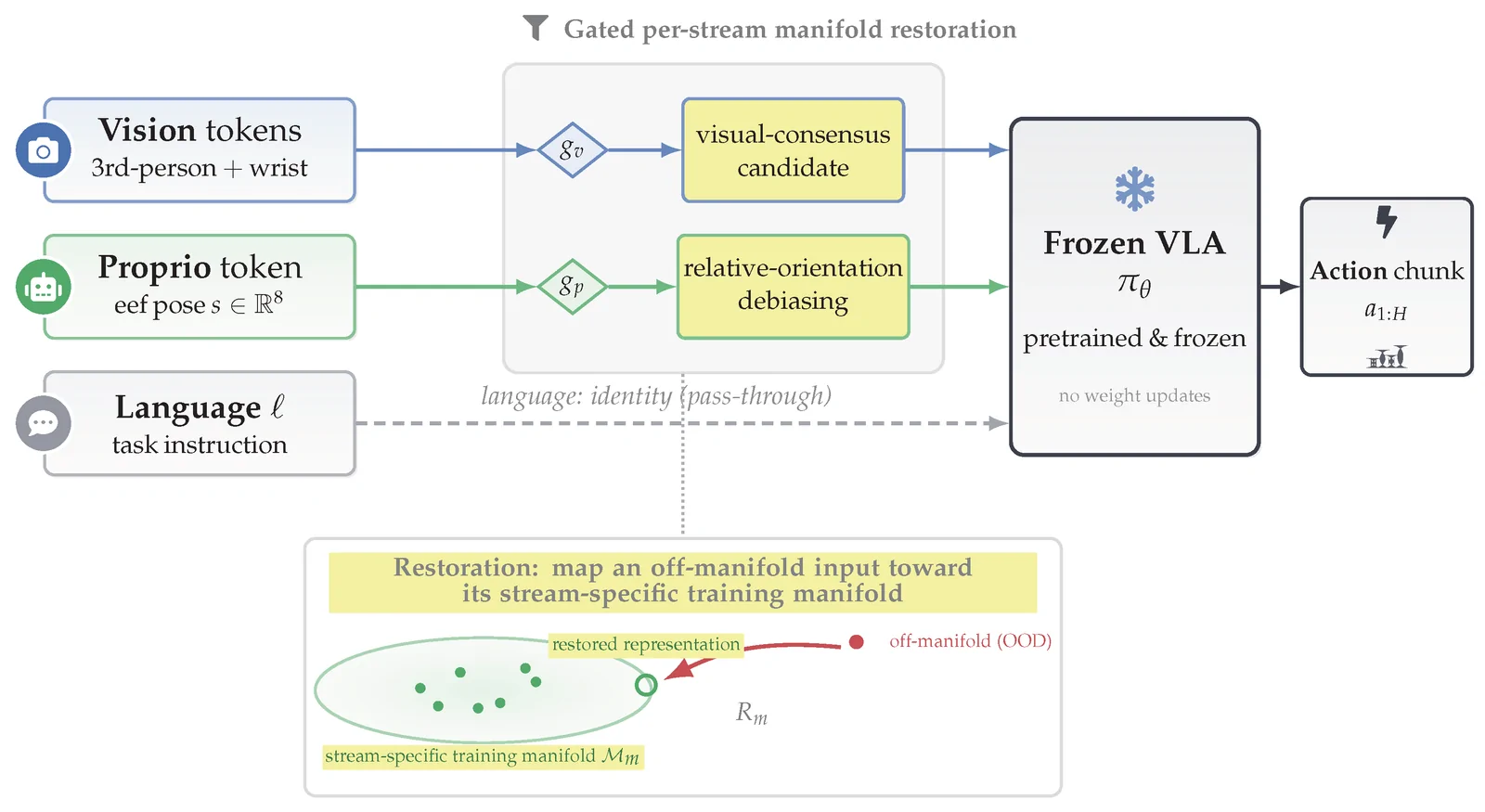
Gated Per-Stream Manifold Restoration (G-PSMR). A frozen Vision–Language–Action policy $\pi_{\theta }$ consumes several input streams. The inset illustrates the generic restoration objective: for stream *m*, $R_{m}$ maps a detected off-manifold input toward the stream-specific training manifold $M_{m}$. For each instantiated restoration, a stream-specific training-free detector determines whether the corresponding input is modified before being consumed by the policy. The evaluated wrapper applies an entropy-gated consensus candidate to the visual stream and relative-orientation debiasing to the proprioceptive stream; the language input passes through unchanged. The policy weights are never updated. The two operators act on disjoint inputs and can be evaluated independently or jointly, allowing their individual effects and cross-stream complementarity to be measured. eef: end effector.

Figure 1 summarizes the proposed per-stream gating and restoration framework.

Our key observation is that VLA out-of-distribution (OOD) failure can be decomposed by input stream. A VLA consumes several modality streams—visual tokens, a proprioceptive state token, and language tokens—each mapped into the backbone embedding space. A viewpoint or appearance shift primarily disturbs the visual stream, whereas a perturbation in the robot’s initial pose directly changes the proprioceptive token. For OpenVLA-OFT, this token is produced by an MLP from the robot state and participates in the same forward pass as the visual and language tokens. Image-space interventions can still influence the policy response indirectly, but they do not explicitly remove the shifted state representation. The blind-test gate audit supports this diagnosis: it activates on 153/160 robot-initial-state instances and on none of the 960 instances from the other six perturbation factors.

We therefore treat test-time robustness as a per-stream restoration problem. The proposed framework, Gated Per-Stream Manifold Restoration (G-PSMR), first checks whether a given input stream is off its training manifold and then applies a restoration only to that stream before the frozen policy consumes it. For vision, entropy-gated test-time augmentation with trimmed-mean action consensus maps unstable visual observations toward a local consensus. For proprioception, gated relative-orientation debiasing removes the episode-level initial-orientation bias while preserving the complete within-episode orientation trajectory. The two operators act on different streams and can therefore be executed independently or jointly. In the original paired evaluation, their joint use improves total success by +5.3 points, compared with a +3.2 image-only gain, and raises robot-initial-state success from 20% to 38%. The new instance-disjoint evaluation independently reproduces the targeted state-side improvement (+5.0 points), and a prospectively specified 600-instance study confirms a +5.8-point gain in the gate-active domain. These results strengthen the central claim that restoration is most effective when its intervention locus matches the stream carrying the shift.

This study’s contributions to the literature are as follows:

- A *stream-decomposition* view of VLA test-time OOD robustness that identifies the input representation affected by a shift and is supported by a selective state-gate audit on the independent test (Section 3.2).
- G-PSMR, a unifying training-free framework that gates a per-stream manifold-restoration operator, including gated relative-orientation debiasing, which preserves the complete within-episode orientation trajectory and requires neither weight updates nor an additional policy forward (Section 3).
- Experiments showing +5.3 points for joint restoration in the original paired evaluation, together with a 1120-instance manifest-disjoint evaluation, a 600-instance prospectively specified confirmation, gate-selectivity analysis, simulator diagnostics, and reproducible qualitative recoveries (Section 4).

## 2. Related Work

**VLA models, robustness benchmarks, and the proprioception problem.** Generalist manipulation policies map images and language to actions at scale, from RT-2 and OpenVLA to Octo and the $\pi_{0}$ flow model [1,4,5,6,7]. A salient architectural distinction underlies our work: RT-2 and OpenVLA incorporate images and language only, whereas OpenVLA-OFT, $\pi_{0}$, and Octo feed the robot state as a *dedicated input token*—in OFT, an eight-dimensional state vector projected by a two-layer MLP into the backbone’s embedding space [2]—making the proprioceptive token a recently introduced, isolatable failure surface. A growing body of work then *measures* the resulting brittleness: LIBERO-Plus perturbs seven factors and finds extreme sensitivity to camera viewpoint and robot initial state [3,8], while VLATest, a multimodal perturbation study, and RoboArena reach similar conclusions and argue that fixed-scene benchmarks overstate capability [9,10,11]. Closest to our diagnosis, recent studies show that visuomotor policies *overrely* on proprioception and generalize poorly—an instance of causal confusion in imitation learning [12]—with fixes that *drop* or *rebalance/retrain* the state input [13,14]; relatedly, multimodal visual RL learns more robust control representations by dissecting shared from modality-specific dynamics [15]. We instead *keep* proprioception and, at test time and in a training-free manner, debias its initial orientation reference while preserving the subsequent relative trajectory.

**Training-free test-time methods for frozen policies.** A complementary deployment constraint is model efficiency: source-data-free pruning compresses pretrained networks without revisiting the original training data [16]. Meanwhile, a large literature improves a frozen model purely at inference—increasingly effective for large multimodal models, from agentic visual grounding and symbolic event reasoning to efficient video LLMs that update no weights [17,18,19]—along three lines with which our work contrasts. *Test-time adaptation* updates weights via entropy minimization or self-supervision [20,21,22] or aggregates over augmentations (MEMO) [23,24]; we keep weights frozen (no gradients), use entropy as a *gate*, and aggregate over augmentations in *action* space via a trimmed mean. *Test-time scaling* samples and *selects* actions (as with RoboMonkey, verifier-free best-of-*N*, and temporal voting) or *detects* failures at runtime (as with Sentinel, FAIL-Detect, SAFE, and FORTRESS) [25,26,27,28,29,30,31]; these methods *select* or *flag* but do not *restore* a corrupted input. *Inference-time intervention* edits hidden activations (as with activation addition, inference-time intervention, and steering of frozen VLAs) or output scores (classifier-free guidance and test-time policy composition) [32,33,34,35,36,37]; we instead intervene at a *third locus, the input stream*, restoring observations and the proprioceptive token *before* the frozen model. Crucially, all three lines act on the visual or action side and leave the proprioceptive failure untouched.

**Input restoration, purification, and canonicalization.** Most directly related, several methods restore a frozen model’s *inputs*. Diffusion purification and source-projection front ends repair corrupted *images* [38,39]; BYOVLA edits distractors on a frozen VLA’s observation [40]; and viewpoint methods canonicalize the camera *view*, whether at training time (GeoAware-VLA) or via test-time novel-view synthesis (AnyCamVLA) [41,42]. The principle that mapping inputs to a canonical pose makes a frozen backbone behave equivariantly is formalized by learned canonicalization [43]. Our visual restoration is in this spirit but avoids generative editing; uniquely, we additionally debias the proprioceptive orientation trajectory relative to its episode start—a state-stream restoration none of these visual methods performs—and gate every restoration to inputs detected to be OOD. The gate limits unnecessary intervention and permits complementary restorations to be combined without updating the policy.

**Manifold projection and out-of-distribution recovery.** Our framing rests on the manifold hypothesis: in-distribution data concentrate near a low-dimensional manifold [44]; therefore, leaving it signals that the data are OOD, as captured by energy-based detectors [45] and a broad open-set/OOD literature spanning few-shot concept-discovery detectors, diversified negative prototypes for few-shot open-set recognition, and real-time multimodal anomaly detection [46,47,48]. Our per-stream gate is a lightweight, training-free instance of such a detector. A natural remedy is to *project* an off-manifold input back onto the data manifold before downstream use, as in Defense-GAN [49,50]. In robotics, “Back to the Manifold” learns a recovery controller that increases state density [51]. We adapt this principle to a frozen multi-stream VLA: instead of learning a recovery policy or a single global projector, we apply a *per-input-stream*, training-free, test-time restoration that projects an off-manifold input toward its training manifold. The original paired evaluation demonstrates complementary visual and state gains, while the instance-disjoint evaluation provides convergent evidence and the prospectively specified confirmation establishes the targeted state-stream effect in the gate-active domain.

## 3. Method

### 3.1. Preliminaries: VLA Policies as Multi-Stream Models

We consider a frozen, pretrained VLA policy $\pi_{\theta }$ for language-conditioned manipulation. At each control step, the policy receives a structured observation o=(I,w,s,ℓ), where *I* is a third-person image, *w* is a wrist image, *s* is a proprioceptive state vector, and *ℓ* is a language instruction. It outputs an *action chunk* $\pi_{\theta }\left(o\right)=\left(a_{1},\ldots ,a_{H}\right)\in R^{H\times 7}$ containing *H* future end-effector deltas, which are executed in an open-loop manner before the next query. In our experiments, $\pi_{\theta }$ is OpenVLA-OFT [2]. The model tokenizes each stream and feeds the resulting sequence to a frozen language-model backbone. We write this factorization as shown in Equation (1):

(1) $$z\left(o\right)=\left[\phi_{v}\left(I,w\right),\;\phi_{p}\left(s\right),\;\phi_{\ell }\left(\ell \right)\right],\;\pi_{\theta }\left(o\right)=h_{\theta }\left(F_{\theta }\left(z\left(o\right)\right)\right),$$

where $\phi_{v}$, $\phi_{p}$, and $\phi_{\ell }$ are the stream tokenizers; $F_{\theta }$ is the frozen backbone; and $h_{\theta }$ is the action head. In OpenVLA-OFT, the proprioceptive vector $s\in R^{8}$ contains the end-effector position, axis-angle orientation, and gripper state; $\phi_{p}$ projects it with a two-layer MLP into one additional input token. The action head is a deterministic $L_{1}$-regression head. As a result, identical inputs produce identical chunks, and any test-time diversity must come from controlled input perturbations rather than output sampling.

### 3.2. Decomposition of OOD Failures by Input Stream

Our starting point is both empirical (Section 4.3) and architectural. Each input stream is mapped into the backbone embedding space, where training exposes the policy to a limited range of token values. Let $M_{m}$ denote the training manifold for stream m∈{v,p,ℓ} after tokenization. A perturbation can move one stream away from its manifold, as formalized in Equation (2):

(2) $$\phi_{m}\left(x_{m}\right)\notin M_{m},\;\phi_{m^{\prime }}\left(x_{m^{\prime }}\right)\in M_{m^{\prime }}\;\;\left(m^{\prime }\neq m\right),$$

and the resulting off-manifold token can affect the whole forward pass through self-attention. LIBERO-Plus perturbations naturally separate along these streams. Camera, background, texture, and sensor-noise shifts mainly affect visual tokens; language rewrites affect language tokens; robot-initial-state perturbations directly alter the proprioceptive token by changing the initial end-effector pose. Image-space interventions can influence the policy response indirectly, but they do not explicitly canonicalize this shifted state representation; this motivates a state-side operator for the robot-initial-state factor.

### 3.3. Gated Per-Stream Manifold Restoration

The decomposition motivates a streamwise intervention. Rather than apply one global correction, we restore each stream independently. Let $x_{v}=\left(I,w\right)$, $x_{p}=s$, and $x_{\ell }=\ell$. For each stream *m*, G-PSMR uses a binary gate $g_{m}$ whose statistic perturbs or measures only stream *m* while holding the other streams fixed, as well as a restoration operator $R_{m}:X_{m}\to X_{m}$, as defined in Equation (3):

(3) $${\tilde{x}}_{m}\;=\;\left\{\begin{array}{cc} R_{m}\left(x_{m}\right) & \mathrm{if}\;g_{m}\left(x_{m}\right)=1\;(\mathrm{stream}\;\mathrm{detected}\;\mathrm{to}\;\mathrm{be}\;\mathrm{off}\;\mathrm{manifold})\\ x_{m} & \mathrm{otherwise}. \end{array}\right.$$

G-PSMR then queries the frozen policy on the restored observation $\tilde{o}=\left({\tilde{x}}_{v},{\tilde{x}}_{p},{\tilde{x}}_{\ell }\right)$. Equivalently, Equation (4) gives the test-time action chunk:

(4) $$\hat{c}=\pi_{\theta }\left({\tilde{x}}_{v},{\tilde{x}}_{p},{\tilde{x}}_{\ell }\right),\;\theta \;\mathrm{fixed}.$$

The formulation above gives the generic per-stream form. The configurations evaluated in this study instantiate restoration operators only for the visual and proprioceptive streams; the language input is passed to the frozen policy unchanged. The gate prevents unnecessary changes to inputs that are already in distribution. Separate gates and operators allow restorations to be matched to different streams and applied jointly without forcing a single shared correction; their individual and joint gains are evaluated in the experiments below.

### 3.4. Visual-Stream Restoration: Entropy-Gated TTA with Trimmed-Mean Consensus

For the visual stream, we restore an unstable observation by aggregating the policy’s responses over a small neighborhood of label-preserving augmentations. The consensus action chunk is used as the policy’s more stable interpretation of the scene.

**Augmentation pool.** At each policy re-query, we construct a single ordered pool ${\left\{T_{i}\right\}}_{i=1}^{n_{\max}}$, with $n_{\max}=8$. The first candidate is deterministically the identity transform, $T_{1}\left(I\right)=I$. For each i=2,…,8, we independently sample a brightness factor $b_{i}∼U\left(0.85,1.15\right)$, a gamma exponent $\gamma_{i}∼U\left(0.85,1.15\right)$, horizontal and vertical translations $\Delta x_{i},\Delta y_{i}∼\mathrm{Unif}\left\{-6,\ldots ,6\right\}$ pixels, and an in-plane rotation $\theta_{i}∼U\left(-4^{{}^{\circ}},4^{{}^{\circ}}\right)$. Each non-identity candidate applies all four operations, in the order brightness, gamma, translation, and rotation; the spatial transforms use border replication. Thus, there is no additional Bernoulli selection probability for individual augmentation types.

The probe and restoration stages use the same frozen distribution rather than separately tuned distributions. The probe evaluates $T_{1}$ and $T_{2}$. If the gate opens, $T_{3},\ldots ,T_{8}$ are additionally evaluated and all eight chunks are passed to the trimmed-mean aggregator; if the gate remains closed, no additional candidates are evaluated and the identity-view chunk is retained. For reproducibility, the reported runs use augmentation seed 0, with each pool deterministically keyed by the suite, task, re-query index, and configuration. Thus, each M1 and M3 visual stream is exactly replayable; small differences between the separately evaluated configurations can still arise from their candidate realizations even when the state gate remains closed.

**Gate.** Because the policy head is deterministic, we estimate uncertainty through disagreement under input augmentation. We draw a cheap probe of $n_{\mathrm{probe}}\;=\;2$ mild augmentations (brightness, gamma, and small translation/rotation) and obtain the action chunks defined in Equation (5):

(5) $$c_{i}=\pi_{\theta }\left(T_{i}\left(I\right),w,s^{⋆},\ell \right),\;i=1,\ldots ,n_{\mathrm{probe}},$$

where the candidate transform is applied only to the third-person image and the wrist image is kept unchanged. Here, $s^{⋆}$ is the current state input: *s* for the visual-only variant and $\tilde{s}$ when proprioceptive restoration is composed first. We compute the normalized mean pairwise chunk distance in Equation (6):

(6) $$u_{v}=\frac{2}{n_{\mathrm{probe}}\left(n_{\mathrm{probe}}-1\right)}\sum_{i<j}\frac{{∥c_{i}-c_{j}∥}_{F}}{\sqrt{7H}}.$$

For a deterministic continuous-action policy, this disagreement plays the role of an entropy surrogate: high disagreement means that small label-preserving visual changes induce unstable actions. Let *t* index policy re-queries within an episode. To obtain an episode-specific and causal reference for action disagreement, we maintain an exponential moving average that is reset at every episode boundary. The first two-view probe warm-starts the estimate: the identity-view chunk is used and ${\bar{u}}_{v,1}=u_{v,1}$. For t≥2, the gate in Equation (7) compares the current disagreement with the pre-update estimate:

(7) $$g_{v,t}\left(I,w|s^{⋆},\ell \right)=1\;\left\{u_{v,t}>\kappa {\bar{u}}_{v,t-1}\right\},\;\kappa =1.0.$$

After the gate decision, the running estimate is updated according to Equation (8):

(8) $${\bar{u}}_{v,t}=\beta {\bar{u}}_{v,t-1}+\left(1-\beta \right)u_{v,t},\;\beta =0.9.$$

The update is applied at every re-query, irrespective of whether the gate opens. Thus, ${\bar{u}}_{v}$ is an episode-local exponential moving average rather than a finite-window statistic, and no state is carried across episodes. When the gate remains closed, the identity-view chunk is retained after the two-view probe; when it opens, the candidate set is expanded to $n_{\max}=8$ and aggregated by the trimmed mean. The values of κ, β, $n_{\mathrm{probe}}$, and $n_{\max}$ were fixed before blind evaluation.

**Restoration.** When the gate opens, we expand to $n_{\max}=8$ augmentations and aggregate the resulting chunks with a per-coordinate *trimmed mean*. Let $C={\left\{c_{i}\right\}}_{i=1}^{n_{\max}}$ be the chunks from the augmented observations, and let $c_{(1),h,k}\leq \cdots \leq c_{(n_{\max}),h,k}$ denote the values sorted for action step *h* and coordinate *k*. With trim ratio ρ=0.2 and $q=\lfloor \rho n_{\max}\rfloor$, Equation (9) defines the restored chunk:

(9) $${\hat{c}}_{h,k}^{\;v}=\frac{1}{n_{\max}-2q}\sum_{i=q+1}^{n_{\max}-q}c_{(i),h,k},\;h=1,\ldots ,H,\;k=1,\ldots ,7.$$

This suppresses outlier augmentations while preserving a coherent chunk. Aggregation is performed in action space and requires no additional model. Because each candidate view requires a policy evaluation, its computational cost depends on the number of candidates; measured inference costs are reported in the inference-cost evaluation. The augmentation family and aggregation rule were selected during the original-study development stage and then frozen before the instance-disjoint blind evaluation.

### 3.5. Proprioceptive-Stream Restoration: Gated Relative-Orientation Debiasing

Robot-initial-state perturbations introduce an episode-level orientation offset before policy execution. The task-relevant signal during control, however, comprises not only the absolute orientation but also its evolution relative to the start of the episode. A restoration that replaces every current orientation with a fixed canonical value would remove this relative motion. We therefore factor the current orientation into an initial reference and a relative trajectory, and correct only the former.

Let $R_{0},R_{t}\in \mathrm{SO}\left(3\right)$ denote the initial and current end-effector orientations, and let $R_{c}$ be an in-distribution reference. We preserve the complete relative trajectory $\Delta R_{t}=R_{0}^{-1}R_{t}$ according to Equation (10):

(10) $${\tilde{R}}_{t}=R_{c}\Delta R_{t}=R_{c}R_{0}^{-1}R_{t}.$$

This transformation changes the episode’s initial orientation reference instead of projecting every state onto a time-invariant pose. In particular, ${\tilde{R}}_{0}^{-1}{\tilde{R}}_{t}=R_{0}^{-1}R_{t}$, and so trajectories with different within-episode rotation histories remain distinct after debiasing; only the initial reference is shifted. The gate in Equation (11) uses the geodesic distance $d_{\mathrm{SO}(3)}\left(R_{0},R_{c}\right)$ and is evaluated once, at the first policy query:

(11) $$g_{p}=1\;\left\{d_{\mathrm{SO}(3)}\left(R_{0},R_{c}\right)\geq \tau \right\},\;R_{p}\left(R_{t}\right)=\left\{\begin{array}{cc} R_{c}R_{0}^{-1}R_{t}, & g_{p}=1,\\ R_{t}, & g_{p}=0, \end{array}\right.\;\tau =0.07.$$

The gate decision remains fixed for the episode. At t=0, the transformed orientation equals $R_{c}$; at later steps, it preserves the full orientation change from the episode start. Position and gripper entries are unchanged. We estimate $R_{c}$ separately for each LIBERO suite from a fixed clean-calibration manifest. For each suite, we enumerate the initial states associated with the original, unmodified LIBERO assets and sample 25 states without replacement using calibration seed 20260721. After the same ten simulator-settling steps used before policy execution, we record their end-effector orientations ${\left\{R_{i}\right\}}_{i=1}^{25}$ and compute the SO(3) chordal $L_{2}$ mean in Equation (12):

(12) $$R_{c}=\arg\min_{R\in \mathrm{SO}(3)}\sum_{i=1}^{25}{∥R_{i}-R∥}_{F}^{2}.$$

The resulting 100-state calibration manifest is constructed without rollout-success or OOD labels and is disjoint from the original-evaluation, blind-test, and confirmation episodes. Thus, the reference is estimated separately for each suite rather than obtained from one episode and shared globally across all suites. This operation remaps only the proprioceptive representation supplied to the frozen policy; it neither moves the robot nor adds a scripted recovery controller.

### 3.6. Joint Application Across Streams

The visual and proprioceptive restorations act on disjoint streams and use independent gates. They can therefore be applied together. In implementation, Equation (13) restores the state stream first:

(13) $$\tilde{s}=\left\{\begin{array}{cc} R_{p}\left(s\right), & g_{p}\left(s\right)=1,\\ s, & g_{p}\left(s\right)=0, \end{array}\right.$$

and the visual gate then decides whether to use the direct chunk $\pi_{\theta }\left(I,w,\tilde{s},\ell \right)$ or the visual consensus chunk from Equation (9). Equation (14) gives the final action chunk:

(14) $$\hat{c}=\left\{\begin{array}{cc} {\hat{c}}^{\;v}, & g_{v}\left(I,w|\tilde{s},\ell \right)=1,\\ \pi_{\theta }\left(I,w,\tilde{s},\ell \right), & g_{v}\left(I,w|\tilde{s},\ell \right)=0. \end{array}\right.$$

This construction enables cross-stream composition while retaining independent gates. We report the visual, state, and joint configurations separately so that both their individual effects and their empirical complementarity can be assessed.

## 4. Experiments

The experiments combine the original paired comparison with two independent evaluations. We retain the 280-episode study that established the original visual, state, and joint results, then evaluate the same three restoration configurations on an instance-disjoint 1120-instance test and use a separate prospectively specified 600-instance study to confirm the targeted state-stream effect. We also report gate selectivity, simulator behavior, inference cost, and qualitative recoveries.

### 4.1. Evaluation Protocol

**Benchmark and policy.** We evaluate the publicly released OpenVLA-OFT checkpoint [2] on LIBERO-Plus [3], which applies seven perturbation factors to the four LIBERO suites [8]: background texture, camera viewpoint, instruction language, lighting, object layout, robot initial state, and sensor noise. The policy is frozen in every configuration.

**Evaluation chronology.** The original 280-episode subset served as the common paired set for the initial comparison and limited configuration development. We therefore treat its results as descriptive evidence from the original study rather than as an independent confirmatory estimate. The restoration operators, gate thresholds, selected configurations, and statistical decision rules were subsequently frozen before the instance-disjoint test outcomes were accessed. Independent statistical claims rely on the 1120-instance evaluation or the separately prospectively specified 600-instance study described below.

**Blind test.** The main test contains 1120 previously unevaluated task instances: seven factors, four suites, and 40 instances per factor–suite cell (n=160 per factor). We define a task instance as one suite-local LIBERO-Plus benchmark entry, uniquely indexed by (suite,task_id). The identifier selects a fixed BDDL task/environment specification and its associated initialization data; the perturbation factor is deterministic metadata of that entry. Two evaluation manifests overlap if and only if they contain the same (suite,task_id) pair (equivalently, the same (suite,factor,task_id) tuple); changing the method or rollout seed does not make a reused benchmark entry a new task instance. The original manifest contains 10 entries per factor–suite cell (280 total; subset-sampling seed 0). We constructed the blind manifest by removing the union of all previously evaluated identifiers, including these 280 original-study identifiers, and then sampling 40 entries without replacement from each remaining factor–suite cell (manifest-sampling seed 20260731). Consequently, the blind and original manifests share 0 identifiers. They retain the same four suites, seven perturbation categories, and potentially the same high-level manipulation goals, but they use different benchmark variants and perturbation realizations (e.g., camera poses, textures, language rewrites, lighting/noise settings, object layouts, or robot start poses). All four configurations are evaluated on the same blind instances, episode seeds, and initial states, producing 4480 paired rollouts: M0 is the frozen policy, M1 is the visual-consensus candidate, M2 is relative-orientation debiasing, and M3 applies M1 and M2 jointly.

**Prospectively specified confirmation.** We additionally evaluate the frozen M0 and M2 configurations on 600 previously unused robot-initial-state instances for which the state gate is active. Before any policy rollout on this manifest, we froze the hypotheses, sampling rule, primary comparison, and analysis. The set is equally stratified across three initial-orientation-offset ranges and four suites (50 instances per offset–suite cell). It estimates the effect within the gate-active domain rather than the natural prevalence or benchmark-wide contribution of that domain.

**Statistics.** All comparisons are paired. We report percentage-point differences, recovered/lost episode counts, exact McNemar tests, and 95% confidence intervals from 20,000 paired bootstrap resamples. The blind campaign applies its prespecified Holm correction across the prespecified comparisons. The prospective confirmation has one primary test.

### 4.2. Original Paired Control Evaluation

**Comparison scope.** In the original study, we evaluated a broad set of locally implemented test-time controls spanning visual restoration, action selection, representation intervention, and uncertainty-based fallback. All controls use the same frozen OpenVLA-OFT checkpoint, paired episode manifest, rollout seed, and success criterion. Their exact operations, fixed settings, and degree of alignment with the cited method families are summarized in Table 1; implementation details are documented in Table A1 of Appendix A.

This paired evaluation provides the original evidence for cross-stream complementarity: M1 improves overall success by +3.2 points, while M3 provides an improvement of +5.3 points and raises RI success from 20% to 38%. The subset contains ten instances per factor–suite cell, and so the following independent evaluations add higher-powered estimates without discarding the completed common- protocol comparison.

### 4.3. Configuration-Frozen Blind Evaluation

The blind evaluation independently identifies the intended targeted state-stream effect (Table 2). M2 improves the robot-initial-state factor from 23.8% to 28.7%, a +5.0-point difference with a paired bootstrap interval of [+0.6,+9.4] and eleven recovered versus three lost episodes. Because robot initial state is one of seven equally weighted factors, the overall change is smaller (71.4%→72.1%). The two-sided exact McNemar test does not pass the prespecified multiplicity-adjusted threshold (unadjusted p=0.057; Holm-adjusted p=0.287).

For completeness, the registered joint comparisons are reported alongside this targeted result. M3 preserves the M2 robot-initial-state success rate of 28.7% and differs from M0 by +5.0 points on RI (CI [−0.6,+10.6]; Holm p=0.231); its overall difference is +0.4 points (95% CI [−1.2,+1.8]; Holm p=0.724). The overall M1–M0 and M3–M2 contrasts are −0.1 points (CI [−1.5,+1.3]) and −0.3 points (CI [−1.7,+1.2]), respectively. The estimated interaction M3−M1−M2+M0 is −0.18 points, with a 95% confidence interval of [−0.89,+0.54]. Thus, the independent campaign directly supports the state-stream mechanism and shows that joint execution preserves its target-factor benefit. The aggregate visual and interaction estimates in this particular blind sample are centered near zero; accordingly, we retain the broader visual and joint gains as findings of the original paired comparison rather than generalize them to every perturbation distribution.

**Figure 2:**
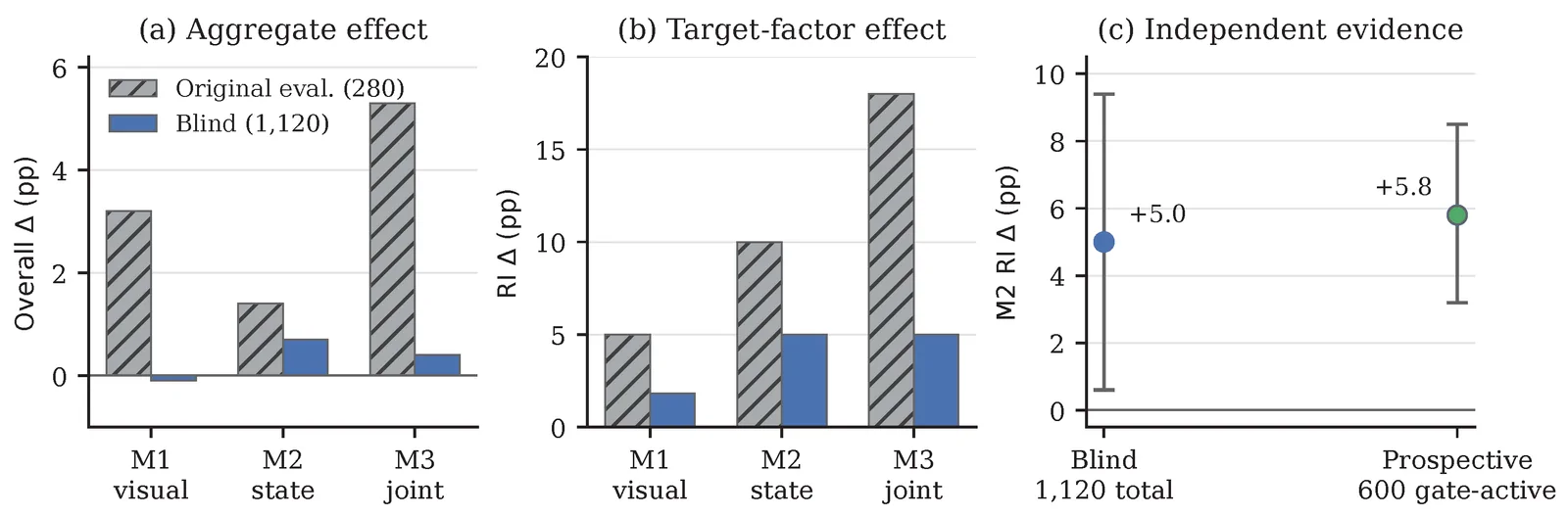
Evidence across the original evaluation, blind evaluation, and confirmation. Panels (**a**,**b**) compare percentage-point differences computed from the displayed original-evaluation and blind-test success rates; hatched original-evaluation bars are descriptive and are not used for confirmatory inference. Panel (**c**) reports the paired 95% confidence intervals for M2 on the robot-initial-state factor in the 1120-instance blind campaign and in the separate 600-instance prospectively specified gate-active confirmation. The panels distinguish the original descriptive results from the independent evidence used for the revised claims.

Figure 2 summarizes the complementary evidence: the original paired evaluation establishes the overall visual and joint gains, the blind comparison shows that M3 retains the M2 target-factor improvement, and the separate confirmation estimates the M2 effect in a prospectively specified gate-active domain.

**Figure 3:**
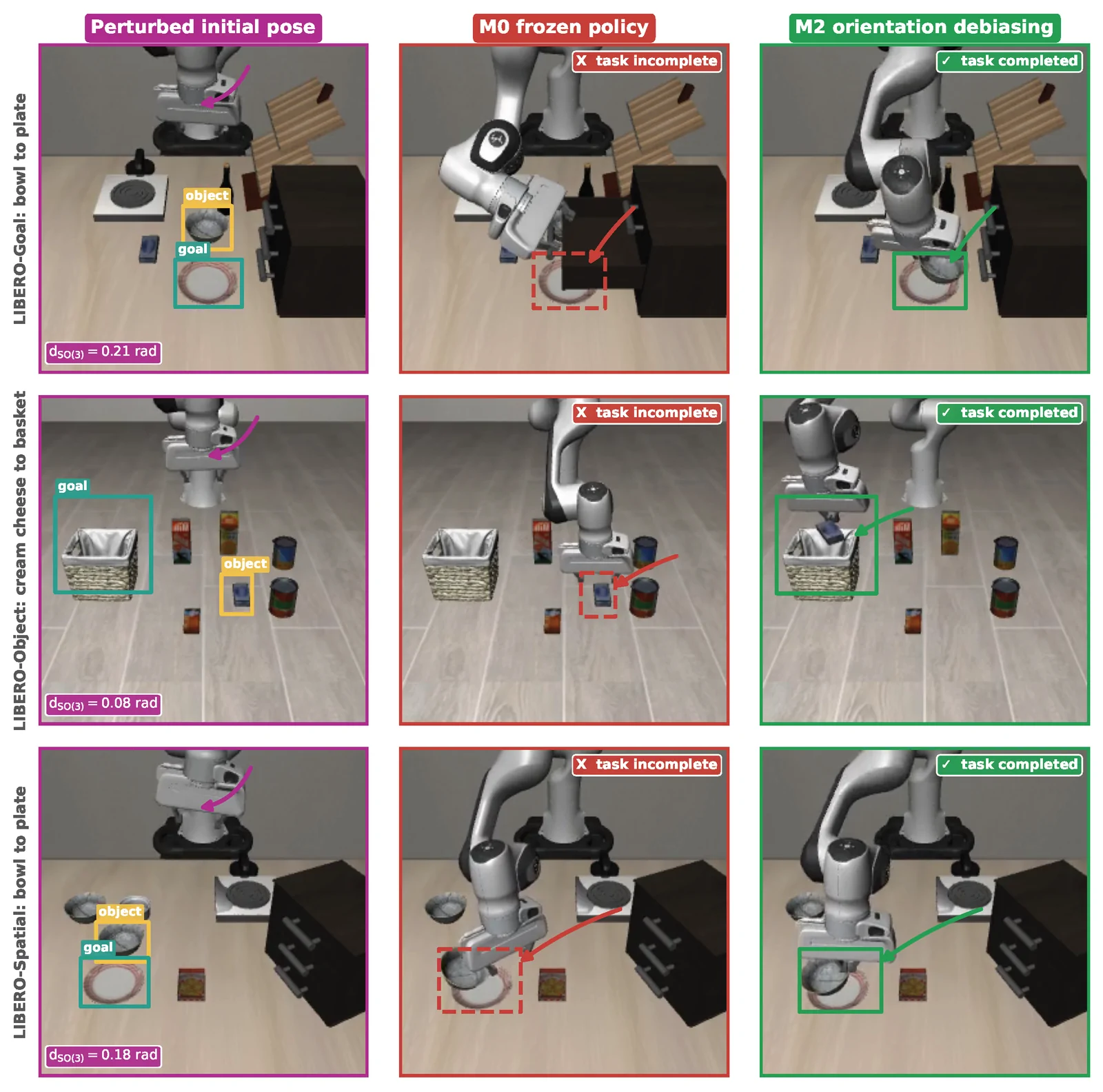
Qualitative state-stream recoveries in the final blind test. Each row shows one instance in which the robot initial state is perturbed, followed by the outcomes of the frozen policy (M0) and relative-orientation debiasing (M2) on the same task. Magenta arrows locate the initial end-effector orientation and report its geodesic offset; yellow and teal boxes mark the manipulated object and goal region. Red and green boxes and arrows call out the incomplete and completed outcomes, respectively. The cases are the first M0-failure/M2-success pair from each suite containing such a pair in frozen manifest order, selected before replay videos were inspected. The replays are illustrative only; Table 2 reports all 160 robot-initial-state instances.

Figure 3 complements these aggregate results with paired examples from the same final blind test. For a reproducible visualization, before inspecting the replay videos we selected the first robot-initial-state instance in frozen manifest order with an M0-failure/M2-success outcome from each suite containing such a pair. The overlays identify the imposed initial end-effector orientation offset, the task-relevant object and goal, and the corresponding M0 and M2 outcomes. A single replay of each selected configuration was used only to capture frames, and all three pairs reproduced their original outcomes; the rates and statistical comparisons above remain those of the original 160-instance blind evaluation.

**Figure 4:**
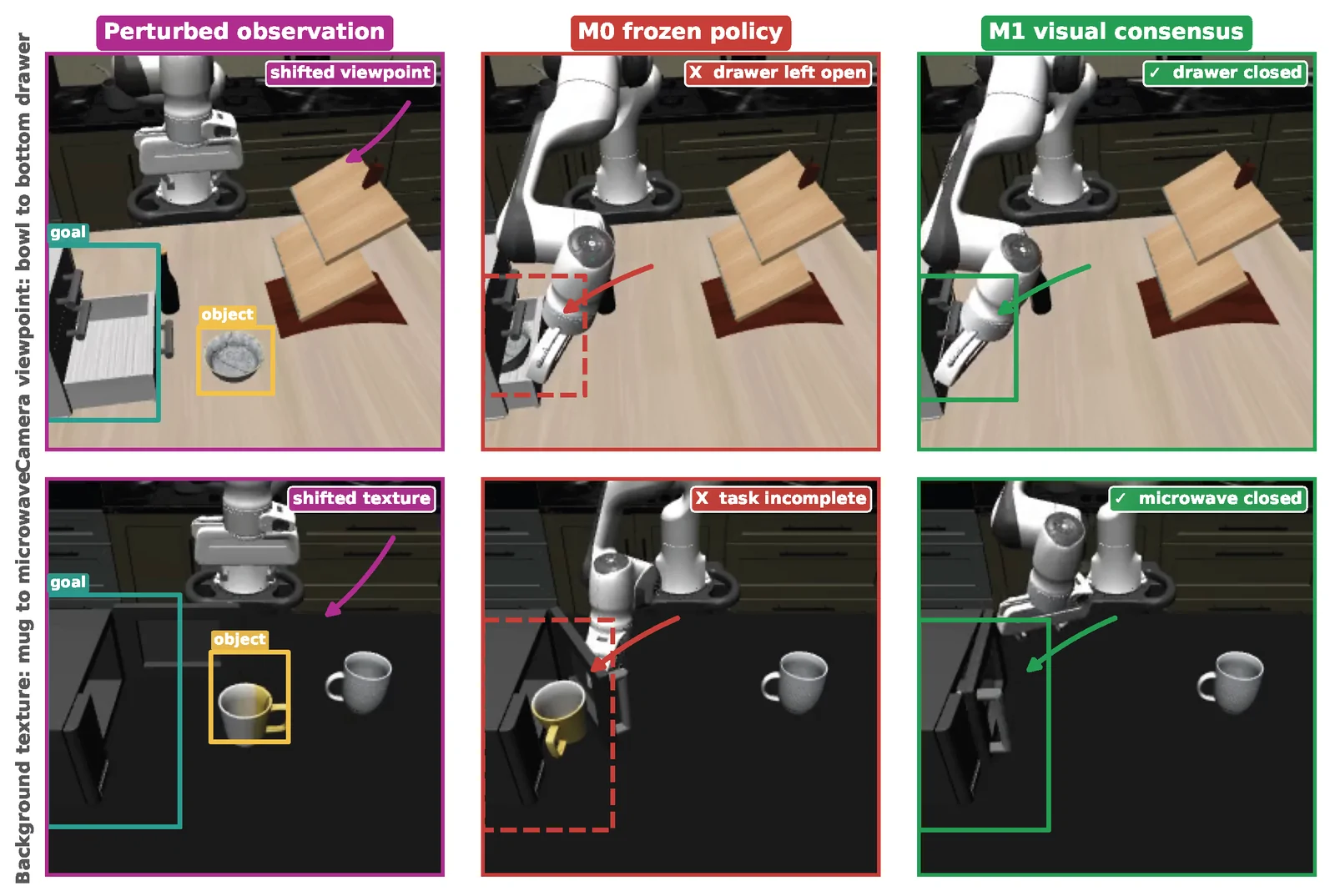
Qualitative visual-stream recoveries in the final blind test. Each row shows a perturbed observation followed by paired M0 and M1 outcomes for the same task. The camera-viewpoint case requires placing a bowl in the bottom drawer and closing it; the background-texture case requires placing a mug in the microwave and closing it. Yellow and teal boxes mark the manipulated object and goal region, and magenta arrows identify the visual shift. Red and green boxes and arrows call out the incomplete and completed outcomes. For each factor, the displayed case is the first M0-failure/M1-success pair in frozen manifest order, fixed before replay videos were inspected. The replays are illustrative only; Table 2 reports all 160 instances for each factor.

Figure 4 provides complementary visual-stream examples for the camera viewpoint and background-texture factors. For each requested factor, we fixed the first M0-failure/M1-success instance in frozen manifest order before inspecting any replay video. In the camera example, M0 leaves the bottom drawer open, whereas M1 completes the placement and closure. In the background example, M0 leaves the mug outside the completed goal state, whereas M1 places it in the microwave and closes the door. Each selected configuration was replayed once only to capture frames, and both outcome pairs reproduced the blind-test results; all rates and statistical comparisons remain those of the original 160-instance-per-factor evaluation.

### 4.4. Prospectively Specified Confirmation of the State-Stream Effect

On the prospectively specified primary comparison, relative-orientation debiasing improves success from 26.3% to 32.2% (+5.8 points; 95% CI [+3.2,+8.5]; 52 recovered versus 17 lost; exact McNemar test $p=2.93\;\times \;10^{-5}$). This independently confirms the targeted effect in the gate-active robot-initial-state domain using the same frozen operator as the blind campaign (Table 3).

The prespecified heterogeneity analyses further locate the effect. The absolute gain is largest in the mild to moderate ranges (+9.0 and +6.5 points), where the underlying tasks remain more recoverable; consequently, the prospectively specified positive dose–response relationship is not observed (Pearson r=−0.071; one-sided permutation p=0.960). Improvements occur for both short- and long-horizon tasks (+7.3 and +4.3 points); their difference is not statistically resolved (95% CI [−2.3,+8.3]).

### 4.5. Gate Selectivity, Simulator Behavior, and Inference Cost

The clean calibration data provide a direct check of the reference assumption. Across the four suites, the largest within-suite P95 geodesic distance to the corresponding reference is 0.0033 rad, and the maximum observed distance is 0.0036 rad, both well below the frozen threshold τ=0.07 rad.

**Reference sensitivity.** We further evaluate whether relative-orientation debiasing depends on a particular clean calibration episode. On the paired 40-instance robot-initial-state subset of the original evaluation, we hold the operator, gate, checkpoint, and all evaluation instances fixed and compare five prespecified single-state reference sets—each using one clean state per suite—with the 25-state per-suite mean used by M2. The five single-state references obtain 22.5–25.0% success (24.5% on average), whereas the multi-state mean obtains 30.0%. The paired differences relative to the mean range from −7.5 to −5.0 points; none is statistically resolved (paired 95% confidence intervals include zero; exact McNemar test p=0.250–0.625). All six references produce the same gate decisions, with activation on 38 of 40 instances, and the largest geodesic distance between a single-state reference and the corresponding mean is 0.00334 rad. The multi-state estimator was fixed independently of this diagnostic analysis and was used unchanged in the blind and confirmation experiments. These results provide no evidence that the reported effect depends on selecting a favorable single calibration episode, but they do not imply universal invariance to the reference estimator.

On the blind test, the state gate activates on 153 of 160 robot-initial-state instances and on none of the 960 instances from the other six factors. Thus, on this benchmark, the intervention targets imposed initial-orientation offsets without altering nominal task instances. This selectivity also explains why M2 changes RI while leaving the other factor rates nearly identical to M0. On the sensor-noise factor, M1 and M3 both obtain 115/160 successes (71.9%) and have identical per-episode outcomes. This agreement is consistent with the state gate remaining closed on all 160 sensor-noise instances, and so the joint configuration adds no state-stream intervention in this condition. The earlier one-episode difference on the 40-instance sensor-noise subset is likewise consistent with candidate-realization variation between separately evaluated configurations rather than a systematic state-restoration penalty.

Table 4 summarizes the simulator-behavior and inference-cost measurements.

M2 produces 2.29 collision events per 1000 control steps, compared with 3.11 for M0, and 75 versus 78 episodes contain a collision. Its external-force P95 is slightly higher (195 versus 189). Neither M0 nor M2 produces an action-saturation event or execution exception. These simulator measurements show a lower observed collision-frequency metric for the state intervention while maintaining stable execution; physical-robot validation remains for future work.

Relative-orientation debiasing retains one policy forward per re-query and has a latency of 124.7 ms, comparable to 122.8 ms for the frozen policy. The visual-consensus candidate averages 4.05 forwards per re-query and reaches 1148.6 ms, while the joint configuration reaches 1156.3 ms. This quantifies the accuracy–computation trade-off of multi-view consensus; importantly, the state intervention confirmed in the prospective study adds neither policy forwards nor material latency.

### 4.6. Interpretation

Across the three evaluations, the evidence consistently supports the per-stream diagnosis. The original paired study shows complementary visual and state gains and the strongest overall result for their joint application. The instance-disjoint study provides convergent evidence, and the prospectively specified study confirms that relative-orientation debiasing benefits the directly shifted state stream, while the gate audit explains why unrelated perturbations remain unchanged. Joint execution preserves this target-factor improvement. Together, these results support architectural composability and show why empirical gains should be reported for each operator and evaluation distribution.

## 5. Conclusions

We introduced a per-stream view of test-time VLA robustness and G-PSMR, a training-free framework that matches gated restoration to the representation carrying a shift. In the original paired evaluation, visual and state restorations provide complementary gains: their joint use improves overall success by +5.3 points and raises robot-initial-state success from 20% to 38%. The expanded evidence supports the central state-stream mechanism: relative-orientation debiasing improves the target factor by +5.0 points on 1120 unseen instances, and a prospectively specified 600-instance study reveals a +5.8-point effect in the gate-active domain. The state gate activates on 153/160 target instances and 0/960 other-factor instances, while the operator retains one policy forward and baseline-level latency. Together, these results support stream-local diagnosis, targeted restoration, and explicit evaluation of cross-stream compositions.

**Limitations and outlook.** The present evidence is specific to OpenVLA-OFT and LIBERO-Plus; cross-policy and real-robot experiments are the next step toward deployment. The suite-level reference is well supported by the compact clean calibration distributions measured here, while tasks with multimodal initial poses may benefit from task-conditioned or mixture-based references. Relative-orientation debiasing preserves relative motion and shows a lower observed simulator collision frequency, but these diagnostics are not a substitute for hardware safety validation.

## 6. Reproducibility

All configurations wrap a frozen, publicly released OpenVLA-OFT checkpoint and modify no weights. The evaluation design, restoration operators, gate thresholds, statistical procedures, and fixed settings are documented in this article and the Appendix. Further information is available from the corresponding author on reasonable request.

## Figures and Tables

**Table 1:** Original paired evaluation of locally implemented test-time controls on 280 episodes. The joint configuration achieves the largest overall improvement and the highest RI success. Independent evaluations of the same G-PSMR configurations are reported in Section 4.3 and Section 4.4. RI denotes robot initial state, and Cam denotes camera viewpoint; implementation details are reported in Appendix A Table A1.

Method	RI	Cam	Overall (%)	Δ (pp)
Frozen policy (OpenVLA-OFT)	20	62	71.1	–
*Visual restoration/test-time augmentation*				
TTA + medoid consensus	18	60	71.4	+0.3
Classical image restoration	28	70	72.5	+1.4
Entropy-gated TTA (medoid)	22	70	73.9	+2.8
M1: entropy-gated TTA (trimmed mean)	25	72	74.3	+3.2
*Action selection/representation intervention*				
Input-augmented best-of-*N*	20	60	70.9	−0.2
Overlapping-chunk temporal voting	22	68	69.6	−1.5
Activation steering	22	65	72.5	+1.4
Geometric view augmentation	20	62	70.0	−1.1
Sensitivity-guided region neutralization	18	20	32.1	−39.0
Uncertainty-gated fallback	25	50	65.7	−5.4
*G-PSMR configurations*				
M2: relative-orientation debiasing	30	62	72.5	+1.4
M3: visual + state	38	72	76.4	+5.3

**Table 2:** Blind evaluation on 1120 previously unevaluated, manifest-disjoint task instances. All entries are success rates expressed as percentages. M1 is the visual-consensus candidate, M2 is relative-orientation debiasing, and M3 is their joint application.

Configuration	Back.	Camera	Lang.	Light	Layout	RI	Noise	Overall
M0: frozen policy	85.0	60.0	85.6	93.8	77.5	23.8	74.4	71.4
M1: visual-consensus candidate	85.0	59.4	86.9	94.4	76.2	25.6	71.9	71.3
M2: relative-orientation debiasing	85.0	60.0	85.6	93.1	77.5	28.7	74.4	72.1
M3: visual + state	85.0	59.4	86.9	94.4	76.2	28.7	71.9	71.8

**Table 3:** Prospectively specified confirmation on 600 new gate-active robot-initial-state instances, equally stratified by suite and initial-orientation offset.

Initial-Orientation Offset	*n*	M0 (%)	M2 (%)	Δ (pp)
[0.07,0.20) rad	200	47.0	56.0	+9.0
[0.20,0.35) rad	200	20.0	26.5	+6.5
≥0.35 rad	200	12.0	14.0	+2.0
Equally stratified total	600	26.3	32.2	+5.8

**Table 4:** Simulator-behavior and inference measurements on the 1120-instance blind test. Latency is the median, across episodes, of the within-episode 95th-percentile re-query latency.

Configuration	Coll./1k Steps	Coll. Episodes	Force P95	Forwards/Re-Query	Latency (ms)
M0: frozen policy	3.11	78	189	1.00	122.8
M1: visual-consensus candidate	2.91	75	226	4.05	1148.6
M2: relative-orientation debiasing	2.29	75	195	1.00	124.7
M3: visual + state	2.30	72	224	4.05	1156.3

## Data Availability

The data presented in this study are available on request from the corresponding author.

## Appendix A. Implementation Scope of Evaluation Controls

Table A1 documents the implementation scope of the controls in Table 1. “Adapted” denotes an interface-level change required by the deterministic, chunk-predicting OpenVLA-OFT policy. “Family control” denotes a common-protocol implementation of the cited intervention principle when the complete external pipeline is not directly compatible with this policy. Citations identify the provenance of the methodologies, and the final column states the exact scope of each comparison.

**Table A1:** Implementation scope and fixed settings of the original-evaluation controls.

Control	Category	Implemented Operation	Fixed Setting	Scope Relative to Cited Family
TTA with medoid consensus	Local	Identity plus photometric–spatial views; medoid action chunk	N=4	Locally defined control
Entropy-gated TTA	Local	Two-view disagreement probe; expand and aggregate only when gated	$N_{\max}=8$; medoid or trimmed mean	Locally defined control
Classical image restoration	Family control	Non-local-means denoising, bilateral filtering, and gray-world normalization	h=5; d=5; $\sigma_{c}=\sigma_{s}=40$	Classical purification operations [38]
Activation steering	Adapted	Add the normalized mean residual difference between clean and corrupted views in one language-model layer	Layer 16; α=4	Direction fixed before paired evaluation [35]
Geometric view augmentation	Family control	Identity plus perspective-and-rotation warps; medoid action chunk	N=4	Inference-time geometric view operations [41]
Input-aug. Best-of-*N*	Adapted	Select the view with the largest action difference between conditional and empty-instruction predictions	N=4; 2N forwards	Input diversity replaces unavailable output sampling [25,26]
Overlapping-chunk temporal voting	Adapted	Group current-step predictions by cosine agreement and average the majority group	History 4; cosine threshold 0.5	Adapted to deterministic action chunks [27]
Sensitivity-guided region neutralization	Family control	Probe a 3×3 grid; retain high-sensitivity cells and blur the remainder	Keep 50%; blur σ=6	Sensitivity localization with deterministic neutralization [40]
Uncertainty-gated fallback	Family control	Two-view disagreement probe, escalation to medoid consensus, and fixed back-off after persistent uncertainty	$N_{\max}=8$; patience 3	Uncertainty detection and fixed fallback mechanism [31]
